# Differential transcriptome analysis of *Sporocytophaga* sp. CX11 and identification of candidate genes involved in lignocellulose degradation

**DOI:** 10.1186/s40643-023-00629-4

**Published:** 2023-01-30

**Authors:** Jiwei Wang, Ying Zhuang, Xianghe Song, Xu Lin, Xiangyi Wang, Fan Yang, Xiaoyi Chen

**Affiliations:** School of Biological Engineering, Dalian Polytechnic University, Ganjingziqu, Dalian, 116034 People’s Republic of China

**Keywords:** Cellulose biodegradation, *Sporocytophaga*, Differential transcriptome analysis, Carbohydrate-active enzymes, Gliding motility

## Abstract

Cellulose is the most abundant renewable bioresources on earth, and the biodegradation and utilization of cellulose would contribute to the sustainable development of global environment. *Sporocytophaga* species are common aerobic cellulose-degrading bacteria in soil, which can adhere to the surface of cellulose matrix and motile by gliding. In this study, a differential transcriptome analysis of *Sporocytophaga* sp. CX11 was performed and a total of 4,217 differentially expressed genes (DEGs) were identified. Gene Ontology enrichment results showed that there are three GO categories related to cellulose degradation function among the annotated DEGs. A total of 177 DEGs were identified as genes encoding carbohydrate-active enzymes (CAZymes), among which 54 significantly upregulated CAZymes were mainly cellulases, hemicellulases, pectinases, etc. 39 DEGs were screened to associate with gliding function. In order to explore unannotated genes potentially related to cellulose metabolism, cluster analysis was performed using the Short-Time Series Expression Miner algorithm (STEM). 281 unannotated genes were predicted to be associated with the initial-middle stage of cellulose degradation and 289 unannotated genes might function in the middle-last stage of cellulose degradation. *Sporocytophaga* sp. CX11 could produce extracellular endo-xylanase, endo-glucanase, FPase and β-glucosidase, respectively, according to different carbon source conditions. Altogether, this study provides valuable insights into the transcriptome information of *Sporocytophaga* sp. CX11, which would be useful to explore its application in biodegradation and utilization of cellulose resources.

**Graphical Abstract:**

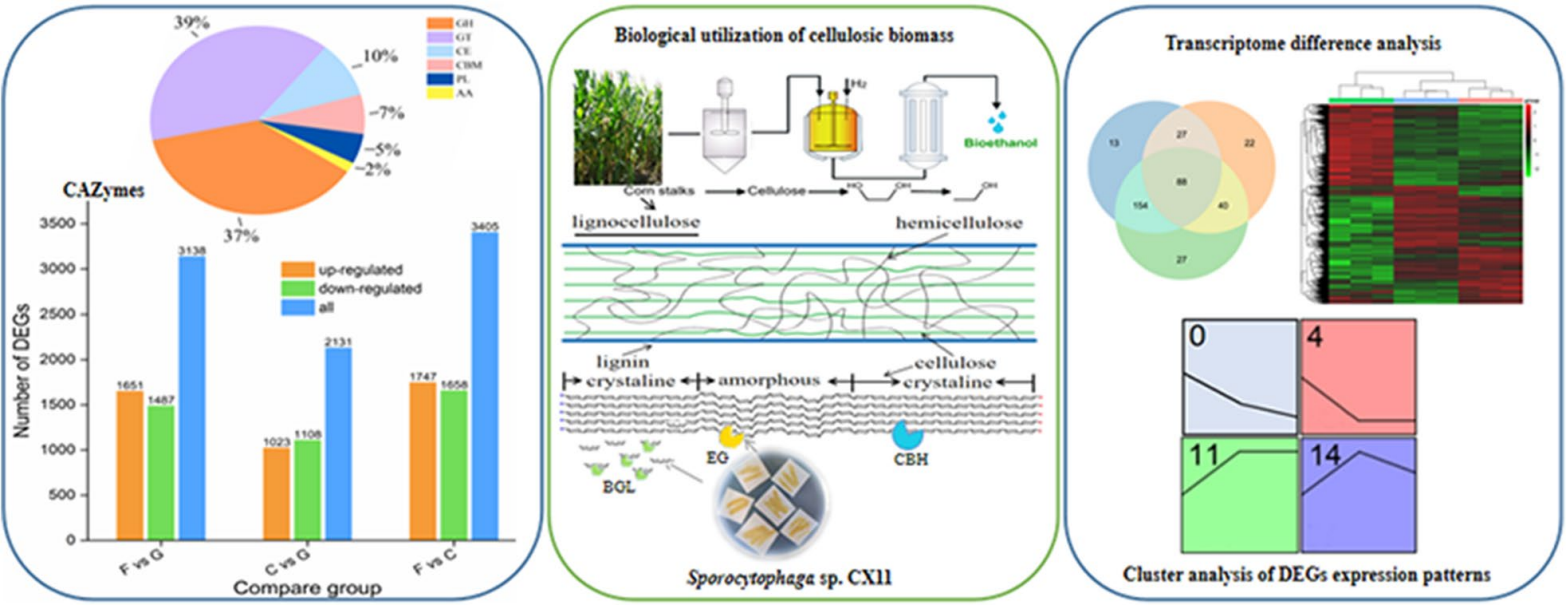

**Supplementary Information:**

The online version contains supplementary material available at 10.1186/s40643-023-00629-4.

## Introduction

Cellulose is the most abundant sustainable bioresource on earth, and can be used to generate biofuels such as ethanol (Lane et al. 2018). Compared with fossil energy, biofuels are renewable and will not cause greenhouse effect. The effective bioconversion and utilization of cellulose matrix is therefore the key to control environmental pollution and achieve both industrial and social sustainability.

As early as in 1919, *Sporocytophaga* species was isolated from soil and determined to be capable of efficiently degrading cellulose (Holt and Leadbetter 1967; Hutchinson and Clayton 1919). *Sporocytophaga*, together with *Cytophaga* (Stanier 1942) and *Sorangium* (Burchard 1981), are kinds of gliding bacteria that are able to strongly adsorbed to the surface of insoluble cellulosic materials to obtain nutrients. The gliding behavior is closely related to their cellulose degradation ability. Strains of the genus *Sporocytophaga* can use cellulose, cellobiose or glucose as the only carbon source and the colonies grown on filter paper are yellow and gummy due to the production of extracellular slime. In 2014, Liu et al. determined the draft genome sequence of *Sporocytophaga* sp. PG-01 (Liu et al. 2014) and mined for genes encoding cellulases and hemicellulases that are closely related to cellulose degradation by the strain. Nonetheless, the studies on the comparative genomics and transcriptomics of *Sporocytophaga* strains have not been reported, and the mechanism of cellulose degradation of them remains to be unclarified.

In previous work, a cellulose-degrading strain designated as CX11 has been isolated and preserved in our lab. According to the polyphasic taxonomy results, strain CX11 should be a new species of the genus *Sporocytophaga* (data not published). In this study, we performed differential transcriptome analysis of *Sporocytophaga* sp. CX11 based on RNA-seq technology and mined genes encoding carbohydrate-active enzymes that play a key role in cellulose degradation of the strain. The experimental and sequencing data generated in this study are supposed to benefit the analysis of cellulose degradation process of strain CX11 and also lay the foundation for the clarification of cellulose degradation mechanism of the genus *Sporocytophaga*.

## Materials and methods

### Bacterial materials and growth conditions

*Sporocytophaga* sp. CX11 was isolated and preserved by our lab at Dalian Polytechnic University. The strain was inoculated on the filter paper double-layer solid medium and cultured at 30 ℃ for 3 days. Then the strain was transferred into liquid medium with filter paper, cellobiose and glucose as the only carbon source, respectively, and cultivated at 30 ℃ and 200 r/min until it reached the early stage of logarithmic phase.

The upper layer of the filter paper double-layer solid medium was Whatman No.1 filter paper and the lower layer was composed of 0.5 g L^−1^ NaNO_3_, 0.5 g L^−1^ MgSO_4_ 7H_2_O, 0.5 g L^−1^ KCl, 1.0 g L^−1^ K_2_HPO_4_ 3H_2_O, 0.01 g L^−1^ FeSO_4_ 3H_2_O, 20 g L^−1^ agar. The composition of the liquid medium was: 5.0 g L^−1^ carbon source, 0.5 g L^−1^ NaNO_3_, 0.5 g L^−1^ MgSO_4_ 7H_2_O, 0.5 g L^−1^ KCl, 1.0 g L^−1^ K_2_HPO_4 _3H_2_O and 0.01 g L^−1^ FeSO_4_ 3H_2_O.

### Total RNA extraction

The total RNA of CX11 cultured under different carbon sources was extracted, respectively, according to the instructions of SteadyPure Universal RNA Extraction Kit (Accurate Biology, China). The extracted RNA samples were checked for quality by agarose gel electrophoresis experiments and Aglient Bioanalyzer 2100 system (Agilent Technologies, USA).

### cDNA library construction and RNA sequencing

RNA samples from cultures with three different carbon sources (nine samples) were submitted to Novogene Co., Ltd. (Beijing) for cDNA libraries construction and Illumina sequencing. Peak plots of sequencing results were converted to raw reads after identification by CASAVA 1.8. Clean reads were obtained by eliminating the low-quality reads (reads containing adapters, reads with quality values lower than 20 and reads containing more than 5% of unclear bases) (Macconaill et al. 2018). The clean reads of nine cDNA libraries were localized to the reference genome of *Sporocytophaga* using Bowtie2 (Langmead and Salzberg 2012), and the localization results were counted to calculate the coverage of the reads on the reference genome. When the ratio was higher than 70%, it indicated that the reference genome was selected appropriately and the sequenced samples were free of exogenous species contamination (Yang et al. 2017).

### Bioinformatics analysis of RNA-seq data

After the sequencing data were processed, the expression levels of differentially expressed genes (DEGs) were calculated using the Fragments Per Kilobase of exon model per Million mapped (FPKM) (Zhao et al. 2021). Differential expression analysis was performed using DESeq package (Wang et al. 2010), and samples from three different carbon sources were compared pairwise. Differential genes were screened with *p* < 0.05 and |log_2_ FC|> 0 as the threshold. The number of upregulated and downregulated genes was subsequently counted according to fold change. Gene Ontology (GO) enrichment analysis of DEGs was implemented by GOseq (Young et al. 2010) to obtain GO categories. The GO function of DEGs was categorized with WEGO (http://wego.genomics.org.cn) (Ye et al. 2006). Hypergeometric test was applied to correct the *p*-value of the enrichment results, and those with *p* < 0.05 were considered as significantly enriched GO categories for DEGs. The Kyoto Encyclopedia of Genes and Genomes (KEGG) pathway analysis of DEGs was performed using KOBAS 2.0 software (Chen et al. 2011). Hypergeometric test was also applied to select metabolic pathways that were significantly enriched.

### Prediction of gene function by short-time series expression miner algorithm

The functions of unannotated genes were predicted based on differential gene expression trends and functional annotations. The unannotated genes with FPKM mean values less than 50 and known functional genes clearly unrelated to cellulose degradation were removed from the three sets of samples, and the remaining genes were regarded as a gene set. The expression pattern analysis was performed using short-time series expression miner (STEM) algorithm (Ernst et al. 2005), and the genes with consistent patterns of expression change were clustered. The unannotated genes that were clustered into the same class with annotated genes were presumed to have similar functions with them.

### Real-time quantitative PCR validation of candidate genes

Real-time quantitative PCR primers were designed by the online design tool (https://sg.idtdna.com/) and Primer Premier 5 (Additional file 1: Table S1). The extracted RNA was reverse transcribed to generate cDNA using the PrimeScript^™^ RT reagent Kit (Perfect Real Time). RT-qPCR was performed using the SYBR Green Polymerase Chain Reaction Kit. Each reaction was performed with a total volume of 20 μL, with a pre-incubation program of 30 s at 95 ℃, followed by 40 cycles of 10 s at 95 ℃, 30 s at 60 ℃ and 15 s at 95 ℃. The housekeeping gene 16S rRNA was used as a reference. PCR procedure for RT-qPCR was performed using standard methods, and the relative expression levels were calculated according to the standard 2^−ΔΔCt^ method (Livak and Schmittgen 2001).

### In situ enzymatic activity assays

To determine the lignocellulose-degrading capacity of strain CX11, enzymatic activity assays of fermentation broth were performed. The total cellulose-degrading activity, endo-1,4-beta-glucanase activity and xylanase activity were tested using Whatman No.1 filter paper, sodium carboxymethyl cellulose (CMC-Na) and xylan (from beechwood) as substrates, respectively. The generated reducing sugars were measured by the method of 3,5-dinitrosalicylic acid (DNS) as described previously (Miller 1959). *p*-Nitrophenyl-β-D-glucopyranoside (*p*NPG) was used as substrate to test the beta-glucosidase activity as reported before (Fang et al. 2014). The fermentation broth was boiled for 30 min to inactivate any possible enzymatic activities, and was used as a negative control. One unit (U) of enzyme activity was defined as the amount of enzyme required to release 1 μM of reducing sugar or *p*NP per minute, and was expressed as U mL^−1^.

### Statistical analysis

All the tests were performed in triplicate unless otherwise stated. Data were expressed as the mean ± standard deviation of triplicate measurements.

## Results and discussion

### Identification and annotation of differentially expressed genes (DEGs)

In the biodegradation of cellulosic substrates, the polysaccharide cellulose is originally hydrolyzed to cellobiose by endoglucanase and/or exoglucanase, which is then converted to glucose by β-glucosidase (Guerrero et al. 2020). It has been reported that the production of cellulases can be induced and regulated by fermentation conditions such as carbon sources, and certain enzymes are only synthesized with the existence of related substrates in the medium (Sukumaran et al. 2021). In this study, we adopted filter paper and cellobiose as the carbon sources, respectively, to induce the production of related cellulose-hydrolyzing enzymes. Glucose was chosen as the third carbon source to represent the situation that none of inducers exist in the medium. Besides, the chosen of the three carbon sources also simulated the process of cellulose degradation. Therefore, the differentially expressed genes under the three types of media should reflect the different roles of genes in cellulose degradation.

**Fig. 1:**
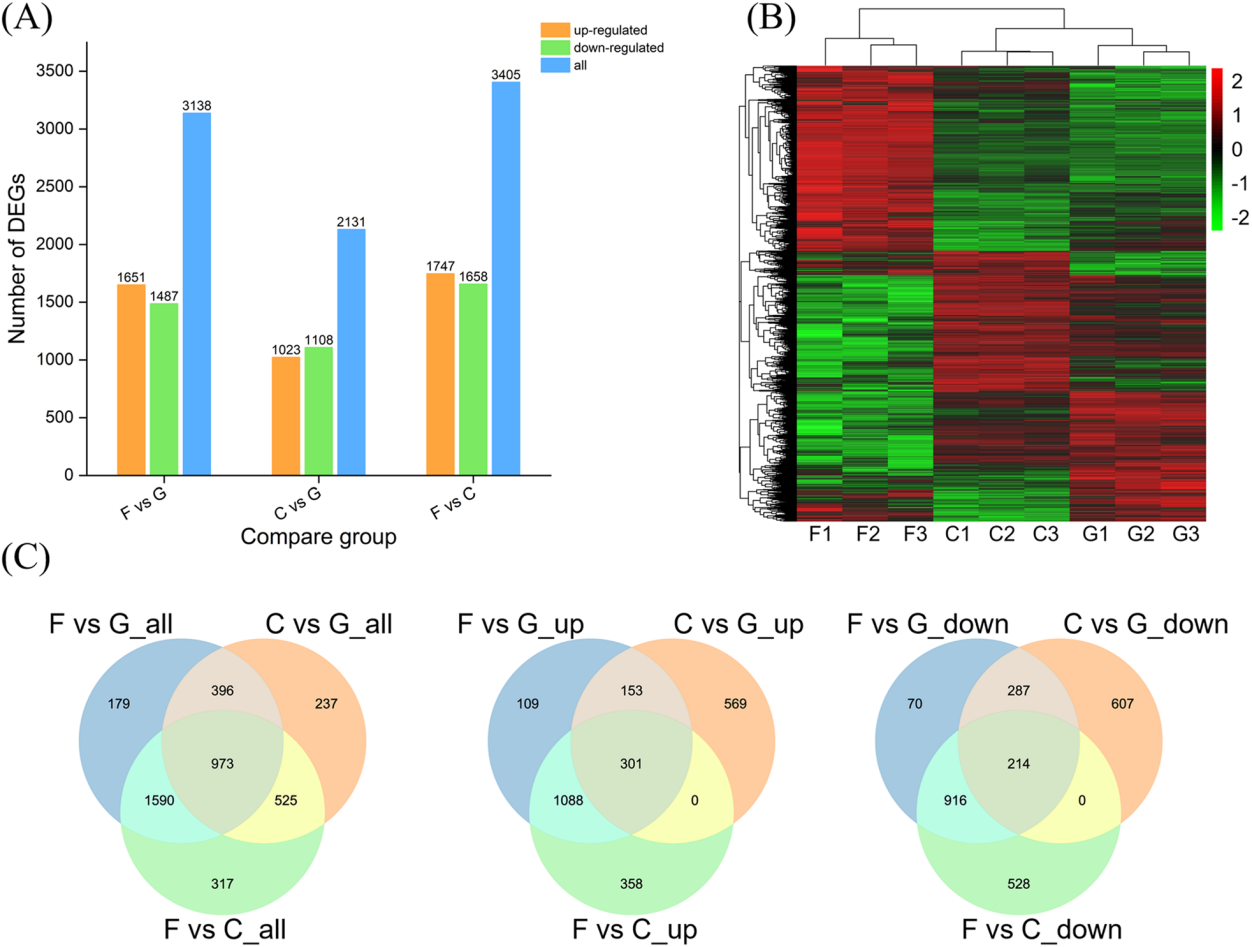
Analysis of DEGs. **A**: Statistical results of DEGs in different carbon source combinations. **B**: Heatmap of clustering of DEGs. **C**: Venn diagram of DEGs

Differential expression genes analysis was performed on FPKM data for three comparative combinations of filter paper and glucose (F vs G), cellobiose and glucose (C vs G), and filter paper and cellobiose (F vs C). The carbon source with higher complexity in each combination was used as the comparison group, and the screening threshold was set to |log_2_ FC|> 0 and padj < 0.05. A total of 4,217 DEGs were differentially expressed in the three combinations, of which a total of 3,138 DEGs were screened in the F vs G combination, 2,131 DEGs were screened in the C vs G combination, and 3,405 DEGs were screened in the F vs C combination ( \* MERGEFORMAT Fig. 1A). The number of up/downregulated DEGs in each combination is displayed in Venn diagram plotting ( \* MERGEFORMAT Fig. 1C), which shows that 973 of the total 4,217 DEGs were identified in all the three combinations. Of these DEGs, 301 genes were upregulated and 214 genes were downregulated. The number of DEGs in the combination with filter paper as the comparison group was significantly greater than that in the combination with cellobiose as the comparison group. These results are consistent with that in previous studies that the complexity of carbon source directly affects the number of DEGs (Gaskell et al. 2016).

The results of clustering analysis of DEGs are shown in \* MERGEFORMAT Fig. 1B. Red color indicates high gene expression and green color indicates low gene expression. Genes in the similar color region were clustered and presumed to have similar functions or participate in regulating the same metabolic pathway. The expression pattern of the same gene in C and G groups is similar, while that in F groups is quite different, indicating that the complexity of carbon source can induce gene expression profile.

### Gene Ontology enrichment analysis of DEGs

**Fig. 2:**
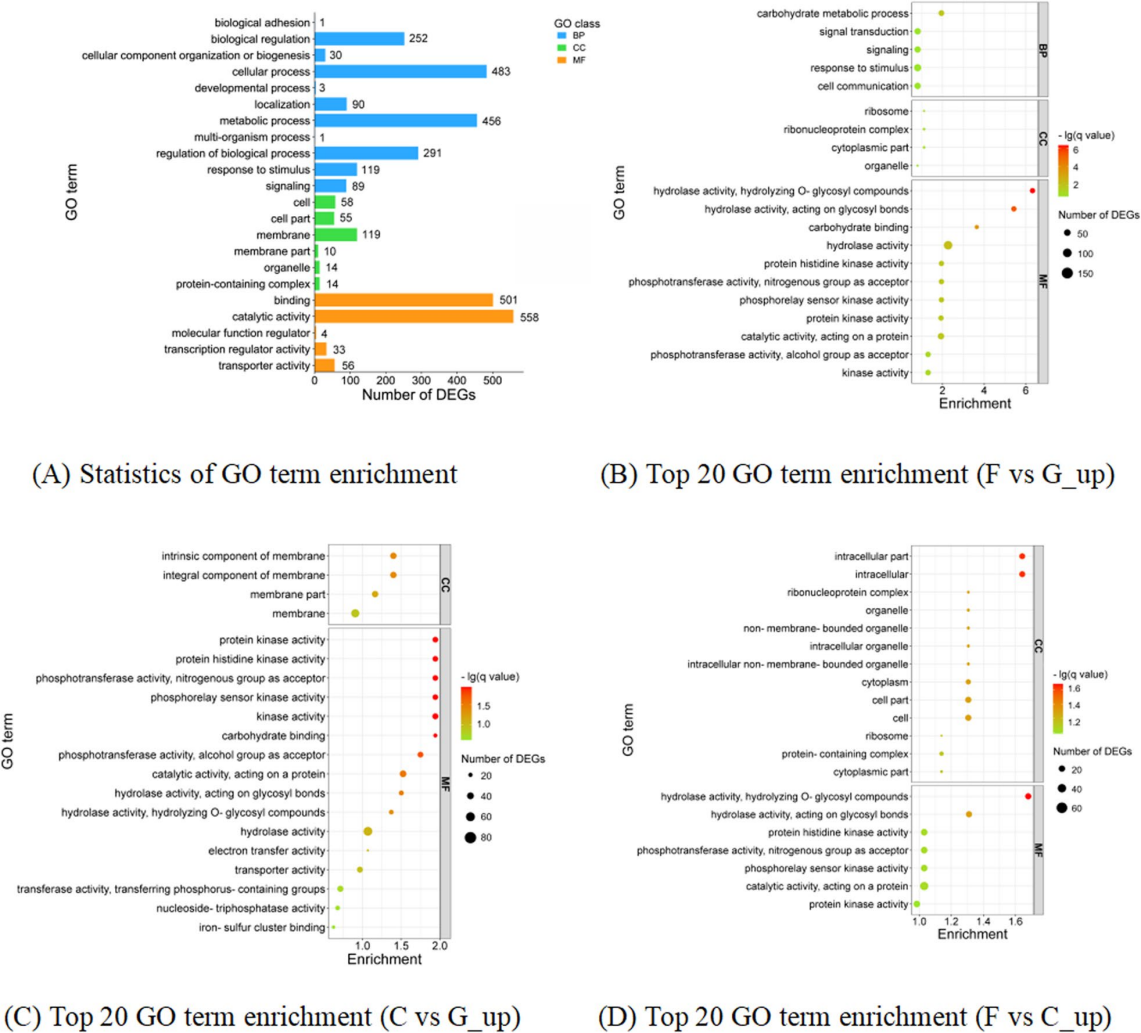
GO enrichment results of DEGs. **A**: Annotation results of total DEGs. **B**: GO significant enrichment results of upregulated DEGs in F vs G combination. **C**: GO significant enrichment results of upregulated DEGs in C vs G combinations. **D**: GO significant enrichment results of upregulated DEGs in F vs C combination. *BP* biological process, *CC* cellular component, *MF* molecular function

To further identify the possible biological functions of DEGs in cellulose degradation, GOseq software was used to perform GO enrichment analysis. A total of 1,589 DEGs were annotated into three major categories: biological process, cellular component and molecular function. In the biological process category, DEGs were mainly enriched on the functional categories such as biological process, metabolic process and cellular process. In the cellular component category, DEGs were mainly enriched on the functional categories such as cell part, membrane and membrane part. In the molecular function category, DEGs were mainly enriched on the functional categories such as catalytic activity, binding, etc. ( \* MERGEFORMAT Fig. 2A).

In this experiment, the number of upregulated DEGs enriched on each functional category was significantly higher than that of downregulated DEGs (Additional file 1: Figures S1-S3). Therefore, the upregulated DEGs were selected for GO significance enrichment analysis (Zheng and Wang 2008). In the biological process category, only carbohydrate metabolic process was significantly enriched (*p* < 0.05) in F vs G_up combination ( \* MERGEFORMAT Fig. 2B). In the cellular component category, no GO categories were significantly enriched in any of the three combinations. In the molecular function category, hydrolase activity acting on glycosyl bonds and O-glycosyl compounds were significantly enriched in all the three combinations. Carbohydrate binding, catalytic activity acting on a protein, and kinase activity were significantly enriched in the F vs G_up ( \* MERGEFORMAT Fig. 2B) and C vs G_up ( \* MERGEFORMAT Fig. 2C).

Based on the Pfam database (Finn et al. 2014) comparison of DEGs enriched in the above categories, a total of three GO categories were found to be associated with cellulose degradation functions, which are carbohydrate metabolic processes, carbohydrate binding and O-glycosyl compound hydrolase activity.

### CAZyme-encoding genes under different carbon sources mapped to the DEGs

**Fig. 3:**
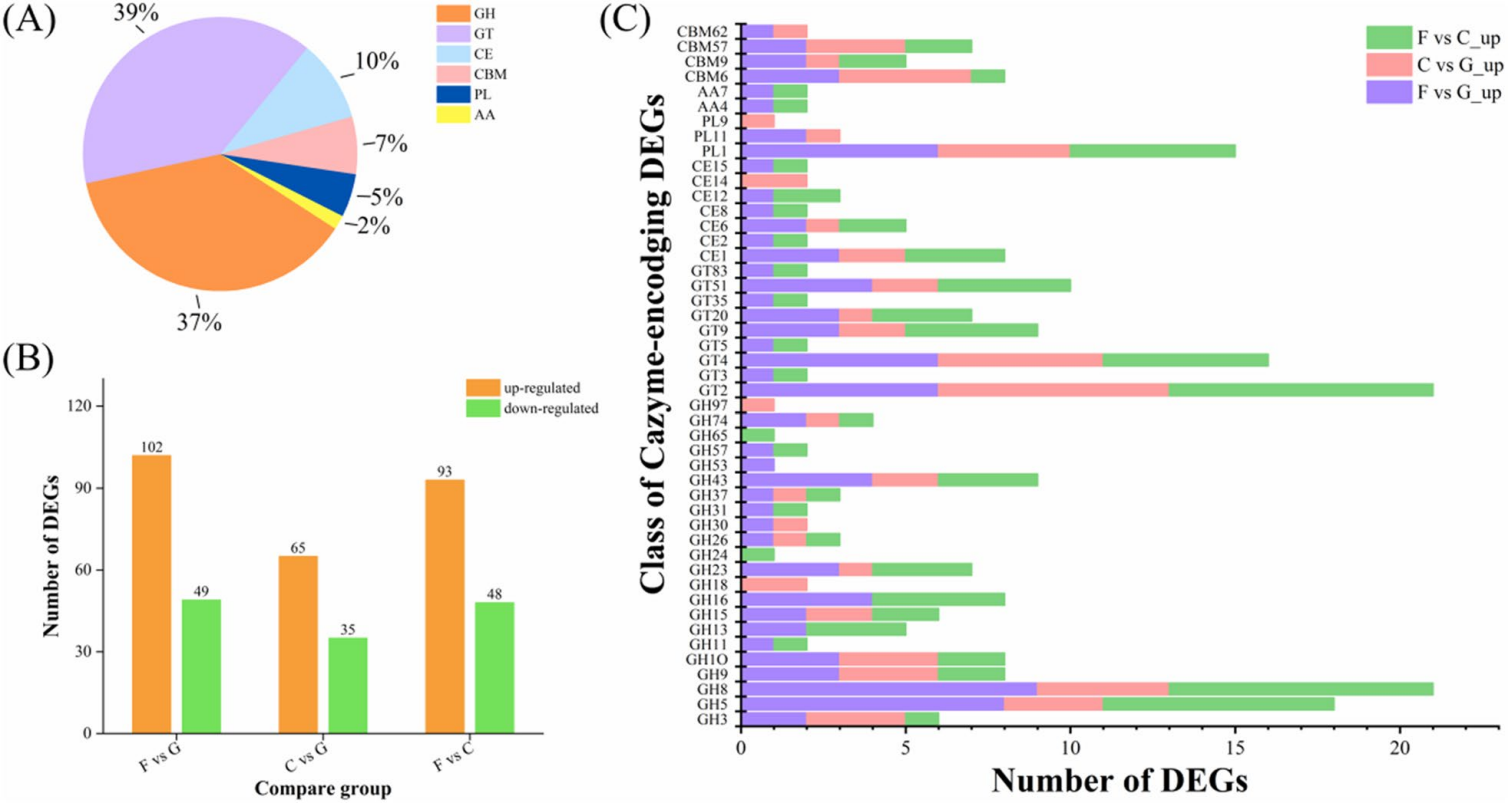
Statistical analysis of DEGs encodingCAZymes. **A**: Family classification of all DEGs. **B**: Statistics of up/downregulated CAZymes in each combination. **C**: Detailed classification of families with upregulated CAZymes in each combination

A total of 177 DEGs were identified as genes encoding carbohydrate-active enzymes (CAZymes), and were classified into six major categories (Drula et al. 2022) according to their annotated functional properties (Fig. 3A). Among them, 66 DEGs were designated in the category of glycoside hydrolases (GHs) and distributed in 22 different families (Fig. 3C). GH8 and GH5 are the two predominant families, in which DEGs were mainly annotated as endoglucanases and reducing-end-xylose releasing exo-oligoxylanases. DEGs in GH43 family were mainly annotated as xylosidases or arabinosidases, while GH9 and GH10 families primarily contain DEGs annotated as endoxylanases and endoglucanases. β-Glucosidases were chiefly distributed in family GH3. These enzymes are all involved in the hydrolyzation of cellulose and hemicellulose. There were 17 DEGs designated in the category of carbohydrate esterases (CEs), and mainly distributed in family CE1 and CE6. Enzymes in these families involved acetylxylan esterases, carbohydrate acetyl esterase/feruloyl esterases, pectinesterases, etc., which usually catalyze the de-O or de-N-acylation of substituted saccharides such as acetylated xylan or pectin methyl esters (Biely 2012). 12 DEGs were annotated as carbohydrate-binding modules (CBMs) and distributed in four different families which are CBM6, CBM57, CBM9 and CBM62. CBMs are a sort of non-catalytic proteins that usually constitute large multi-module enzymes as a relatively independent module, and have been reported to promote enzymatic activity by increasing the effective enzyme concentration on the polysaccharide surface (Boraston et al. 2004).

In the F vs G combination, a total of 151 CAZymes were identified, of which 102 were upregulated and 49 were downregulated. A total of 100 CAZymes with 65 upregulated and 35 downregulated were identified in the C vs G combination. In the F vs C combination, a total of 141 CAZymes were identified, with 93 upregulated and 48 downregulated ( \* MERGEFORMAT Fig. 3B). The statistical data showed that the CAZymes encoding genes with upregulated expression account for more than 65% in all the three combinations, indicating that these enzymes are induced with the existence of filter paper or cellobiose as substrates in the culture medium. A finer classification of the upregulated CAZymes in each combination was performed. As shown in \* MERGEFORMAT Fig. 3C, the upregulated CAZymes were mainly distributed in the GH family, accounting for 48% (F vs G_up), 43% (C vs G_up) and 49% (F vs G_up), respectively. By adjusting the screening threshold to |log_2_ FC|≥ 1 and *p* ≤ 0.01, 54 significantly upregulated carbohydrate-active enzymes were further screened, mainly including cellulases, hemicellulases, pectinases, etc. (Gouvêa et al. 2018) ( \* MERGEFORMAT Table 1).

**Table 1:** Summary of CAZyme-encoding genes with significantly increased expression in groups

Class	Gene ID	Enzyme description	CAZymes family	log_2_ fold change ≥ 1; padj ≤ 0.01		
				F vs G_up	C vs G_up	F vs C_up
Cellulases	*CX11_GM000268*	Beta-glucosidase	GH3	1.02	0.78	
	*CX11_GM004302*	Beta-glucosidase	GH3		1.89	
	*CX11_GM002852*	Endoglucanase	GH5	3.45	0.74	2.59
	*CX11_GM004438*	Endoglucanase	GH5	3.37		3.11
	*CX11_GM002804*	Endoglucanase	GH5	2.92		3.58
	*CX11_GM004508*	Endoglucanase	GH5	1.51	1.55	
	*CX11_GM003220*	Endoglucanase 3	GH5	2.9		2.62
	*CX11_GM001350*	Endoglucanase B	GH5		2.09	
	*CX11_GM000434*	Endoglucanase C	GH5	2.11		1.98
	*CX11_GM003239*	Endoglucanase 2	GH8	3.58	0.96	2.46
	*CX11_GM000388*	Endoglucanase 2	GH8	2.71		2.38
	*CX11_GM001809*	Endoglucanase 2	GH8	2.33	0.41	1.81
	*CX11_GM005339*	Endoglucanase 2	GH8	1.42	1.15	
	*CX11_GM000618*	Endoglucanase C	GH8	2.65		2.51
	*CX11_GM005080*	Endoglucanase A	GH9	1.09	0.48	0.5
	*CX11_GM000056*	Endoglucanase D	GH9	2.34		3.23
	*CX11_GM000079*	Beta-glucanase	GH16	1.33		1.18
	*CX11_GM000626*	Beta-glucanase	GH16	1.07		0.80
Hemicellulases	*CX11_GM000042*	Bifunctional acetylxylan esterase	CE1	2.62	1.44	1.07
	*CX11_GM004263*	Carbohydrate acetyl esterase/feruloyl esterase	CE1	1.18		1
	*CX11_GM001962*	Carbohydrate acetyl esterase/feruloyl esterase	CE6	2.15	1.23	0.81
	*CX11_GM003964*	Carbohydrate acetyl esterase/feruloyl esterase	CE6	2.97		1.84
	*CX11_GM001963*	Mannan endo-1,4-beta-mannosidase	GH5	2.92		2.51
	*CX11_GM000038*	Reducing-end-xylose releasing exo-oligoxylanase	GH8	2.91		2.43
	*CX11_GM002090*	Reducing-end-xylose releasing exo-oligoxylanase	GH8	2.74		2.27
	*CX11_GM000628*	Reducing-end-xylose releasing exo-oligoxylanase	GH8	2.60		1.68
	*CX11_GM005472*	Xyloglucan-specific endo-beta-1,4-glucanase	GH9	0.92	2.42	
	*CX11_GM000081*	Endo-1,4-beta-xylanase B	GH10	2.87	1.06	1.69
	*CX11_GM003219*	Endo-1,4-beta-xylanase B	GH10	2.02	0.93	0.97
	*CX11_GM000047*	Endo-1,4-beta-xylanase B	GH10	1.71	1.31	
	*CX11_GM000039*	Endo-1,4-beta-xylanase 3	GH11	1.38		1.04
	*CX11_GM002851*	Endo-1,4-beta-galactosidase	GH16	2.64		2.86
	*CX11_GM004712*	Mannan endo-1,4-beta-mannosidase	GH26	3.86	0.79	2.95
	*CX11_GM000073*	Glucuronoxylanase	GH30	1.08	0.71	
	*CX11_GM004262*	Arabinoxylan arabinofuranohydrolase	GH43	1.87		1.27
	*CX11_GM000043*	Xylosidase/arabinosidase	GH43		1.35	
	*CX11_GM004261*	Beta-xylosidase	GH43	1.48		1.34
	*CX11_GM000048*	Alpha-l-arabinofuranosidase	GH43	1.19	1.06	
	*CX11_GM002260*	Xyloglucanase	GH74	3.79		3.62
	*CX11_GM000027*	Xyloglucanase	GH74	1.85	1.57	
	*CX11_GM000529*	Mannosyl transferase	GT83	2.00		2.03
Pectinases	*CX11_GM001947*	Pectinesterase 31	CE8	2.9		2.67
	*CX11_GM003962*	Rhamnogalacturonan acetylesterase	CE12	1.56		0.99
	*CX11_GM000527*	l-arabinose transferase	GT2	5.8	1.06	4.62
	*CX11_GM004710*	Pectate lyase E	PL1	2.85	2.14	0.6
	*CX11_GM000632*	Pectate trisaccharide-lyase	PL1	2.3	1.11	1.08
	*CX11_GM000058*	Pectate lyase C	PL1	3.23		1.57
	*CX11_GM003503*	Pectate lyase C	PL1	2.99	1.55	2.16
	*CX11_GM003502*	Pectate trisaccharide-lyase	PL1	1.96	0.68	1.18
	*CX11_GM003504*	Pectate trisaccharide-lyase	PL1	1.49		
	*CX11_GM003956*	Rhamnogalacturonan endolyase	PL11	1.58	1.22	
	*CX11_GM003955*	Rhamnogalacturonan endolyase	PL11	1.07		
Chitinases	*CX11_GM002492*	Chitinase	GH18		1.52	
	*CX11_GM002954*	Chitinase	GH18		1.02	

Most aerobic cellulolytic microorganisms degrade cellulose by secreting a series of free cellulases, while most anaerobic cellulolytic microorganisms perform the degradation process via cellulosome, a large multienzyme complex which bounds to the outer surface of the microorganisms (Wilson 2009; Bayer et al. 2004). In both strategies, synergistic action of endoglucanases (EC 3.2.1.4), exoglucanases (EC 3.2.1.91) and β-glucosidases (EC 3.2.1.21) is required to achieve effective degradation (Zhang et al. 2006). Recently, a few cellulolytic bacteria such as *Cytophaga hutchinsonii* and *Fibrobacter succinogenes*, have been proposed to degrade cellulose by a third strategy in which the bacteria neither secret free cellulases nor possess any cellulosomes (Suen et al. 2011; Zhao et al. 2020). The mechanism of this novel strategy still remains unclarified. Moreover, analysis of genomic sequence of *Cytophaga hutchinsonii* revealed no obvious homologues of known exoglucanases which are generally thought to be critical for the degradation of crystalline cellulose (Xie et al. 2007). As shown in \* MERGEFORMAT Table 1, endoglucanases (mainly distributed in GH5, GH8 and GH9 families) and β-glucosidases (mainly distributed in GH3 family) were synergistically upregulated. However, no exoglucanase or polysaccharide monooxygenase encoding genes (Taillefer et al. 2018) were identified in the genomic database of strain CX11 (data unpublished). Besides, the strain CX11 digests cellulose in a contact-dependent manner which is similar to that of *Cytophaga hutchinsonii*. Therefore, we speculated that *Sporocytophaga* sp. CX11 might also use the third novel strategy mentioned above to degrade cellulose (Zhu and McBride 2017).

### Mining of DEGs related to gliding motility

**Fig. 4:**
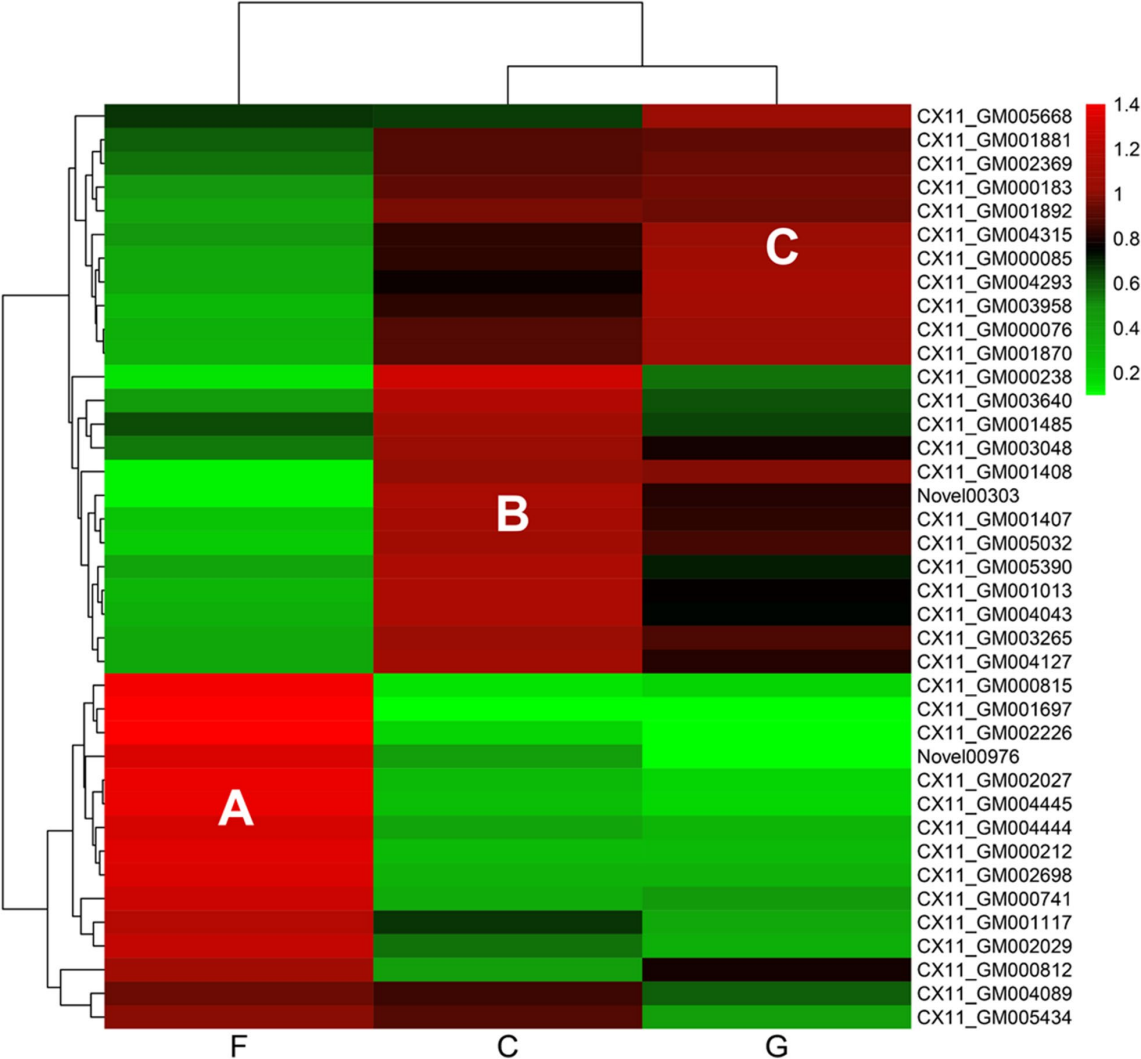
Heat map of clustering of differentially expressed gliding function-related genes. (F/C/G: fermentation supernatant using filter paper, cellobiose and glucose as the sole carbon source, respectively.)

*Sporocytophaga* sp. CX11 is closely related to *Cytophaga hutchinsonii* and exhibits many similarities in metabolism and lifestyle, such as the mode of contact-dependent degradation and rapid gliding motility over surfaces. It has been reported that *C. hutchinsonii* has orthologs for each of the core gliding motility genes of *Flavobacterium johnsoniae*, and the ability to glide over surfaces may allow cells to migrate along cellulose fibers in search of amorphous regions that are most amenable to enzymatic attack (Suen et al. 2011; Xie et al. 2007). *sprP* deletion mutant of *C. hutchinsonii* was partially defective in gliding motility and failed to digest crystalline cellulose, suggesting a link between gliding motility and cellulose utilization (Zhu and McBride 2014). Therefore, DEGs related to gliding motility were also investigated in this study. The differential gene sequences were uploaded to the Pfam database for comparison, and 39 DEGs were screened to associate with gliding function (Additional file 1: Table S2). These genes encode three classes of gliding proteins, including Gld (Glding motility) proteins, Spr (Surface protein) proteins and Sus (Starch utilization system) proteins. The Gld proteins are directly related to the gliding behavior of cells (Mcbride et al. 2009; Rhodes et al. 2011). The Spr proteins are a class of cell surface proteins that can transmit kinetic substances to support cells’ gliding motility (Sato et al. 2021). The Sus proteins mainly participate in the binding and transporting of oligosaccharides, which are important in the digestion of extracellular carbohydrates (Anderson and Salyers 1989). The clustering of expression levels of differential gliding genes under different carbon sources is shown in \* MERGEFORMAT Fig. 4. Under each carbon source condition, there are corresponding high expressed gliding genes, indicating that these gliding genes act at different stage of cellulose degradation.

### Short-time series expression miner algorithm explores more cellulolytic enzymes from *Sporocytophaga* sp. CX11

Although functional annotations of DEGs have been performed through databases such as GO, KEGG and CAZy, there were still a large number of genes whose functions cannot be annotated. To further explore these unannotated genes potentially related to cellulose metabolism, cluster analysis was performed using the STEM algorithm based on different abundance variation patterns of genes, according the procedures proposed by (Yang et al. 2021). Based on the abundance variation patterns, functionally unannotated genes were clustered together with annotated cellulolytic genes and were thus assigned a certain function similar to those of annotated cellulolytic genes from the same pattern. In this study, a total of 16 patterns of gene expression changes were obtained (Additional file 1: Figure S4). Among them, four typical patterns (0, 4, 11 and 14), in which more than 30 genes with annotated functions, were selected for clustering (Table 2).

**Table 2:** Differentially expressed genes belong to 4 different expression patterns

Expression changing pattern	Gene ID	CAZy family	Annotation or putative information
Top 10 differential genes belong to difference changing pattern 0			
	*CX11_GM003764*	NA	Related to the initial and middle phase of cellulose degradation
	*CX11_GM004191*	NA	Related to the initial and middle phase of cellulose degradation
	*CX11_GM000840*	NA	Related to the initial and middle phase of cellulose degradation
	*CX11_GM003150*	NA	Related to the initial and middle phase of cellulose degradation
	*CX11_GM005552*	NA	Related to the initial and middle phase of cellulose degradation
	*CX11_GM002226*	NA	Type IX secretion system membrane protein PorP/SprF
	*CX11_GM004712*	GH26	Endo-1,4-beta-mannosidase
	*CX11_GM002852*	GH5	Endoglucanase
	*CX11_GM000081*	GH10	Endo-1,4-beta-xylanase B
	*CX11_GM000628*	GH8	Reducing-end-xylose releasing exo-oligoxylanase
Top 10 differential genes belong to difference changing pattern 4			
	*CX11_GM004329*	NA	Related to the initial and middle phase of cellulose degradation
	*CX11_GM000033*	NA	Related to the initial and middle phase of cellulose degradation
	*CX11_GM000661*	NA	Related to the initial and middle phase of cellulose degradation
	*CX11_GM000293*	NA	Related to the initial and middle phase of cellulose degradation
	*CX11_GM004381*	NA	Related to the initial and middle phase of cellulose degradation
	*CX11_GM001697*	NA	Type IX secretion system membrane protein PorP/SprF
	*CX11_GM002804*	GH5	Endoglucanase
	*CX11_GM000815*	NA	Gliding motility protein GldM
	*CX11_GM004438*	GH5	Endoglucanase
	*CX11_GM003220*	GH5	Endoglucanase 3
Top 10 differential genes belong to difference changing pattern 11			
	*CX11_GM003811*	NA	Related to the middle and last phase of cellulose degradation
	*CX11_GM003111*	NA	Related to the middle and last phase of cellulose degradation
	*CX11_GM005704*	NA	Related to the middle and last phase of cellulose degradation
	*CX11_GM005293*	NA	Related to the middle and last phase of cellulose degradation
	*CX11_GM000988*	NA	Related to the middle and last phase of cellulose degradation
	*CX11_GM004267*	GH5	Endo-1,4-beta-mannosidase
	*CX11_GM001870*	NA	Type IX secretion system membrane protein PorP/SprF
	*CX11_GM001892*	NA	Type IX secretion system membrane protein PorP/SprF
	*CX11_GM003799*	GH3	Beta-glucosidase
	*CX11_GM003851*	CBM9	Modules of ~ 170 residues found in xylanases and cellulase
Top 10 differential genes belong to difference changing pattern 14			
	*CX11_GM000997*	NA	Related to the middle and last phase of cellulose degradation
	*CX11_GM000199*	NA	Related to the middle and last phase of cellulose degradation
	*CX11_GM001026*	NA	Related to the middle and last phase of cellulose degradation
	*CX11_GM002155*	NA	Related to the middle and last phase of cellulose degradation
	*CX11_GM000995*	NA	Related to the middle and last phase of cellulose degradation
	*CX11_GM002492*	GH18	Chitinase
	*CX11_GM002954*	GH18	Chitinase
	*CX11_GM004043*	NA	Gliding motility lipoprotein GldH
	*CX11_GM003048*	NA	Type IX secretion system membrane protein PorP/SprF
	*CX11_GM005379*	GH3	Beta-glucosidase

The significant patterns of 0, 4, 11 and 14 with more than 30 annotated cellulolytic genes were displayed, and the top 5 of relative abundance of putative cellulolytic genes and candidate cellulolytic genes were listed, respectively

Pattern 0 and 4 are both downward patterns, with all genes in these patterns being expressed at relatively high levels under filter paper conditions. Most of the genes with known functions are annotated as endoglucanases, xylanases and gliding proteins. Therefore, the 281 unannotated genes in pattern 0 and 4 were presumed to be associated with the decomposition of polysaccharides such as cellulose and xylan, which usually own a high degree of polymerization. Pattern 11 and 14 are both upward patterns, and all genes in these patterns are expressed at relatively high levels under cellobiose or glucose conditions. Most of the annotated genes are β-glucosidases and some gliding proteins, suggesting that the 289 unannotated genes in pattern 11 and 14 might function mainly in the degradation of cellobiose or other cellulosic substrates with lower degree of polymerization and basically existed in the hydrolysates of cellulosic polysaccharides.

### Validation of the transcriptome data by RT-qPCR

**Fig. 5:**
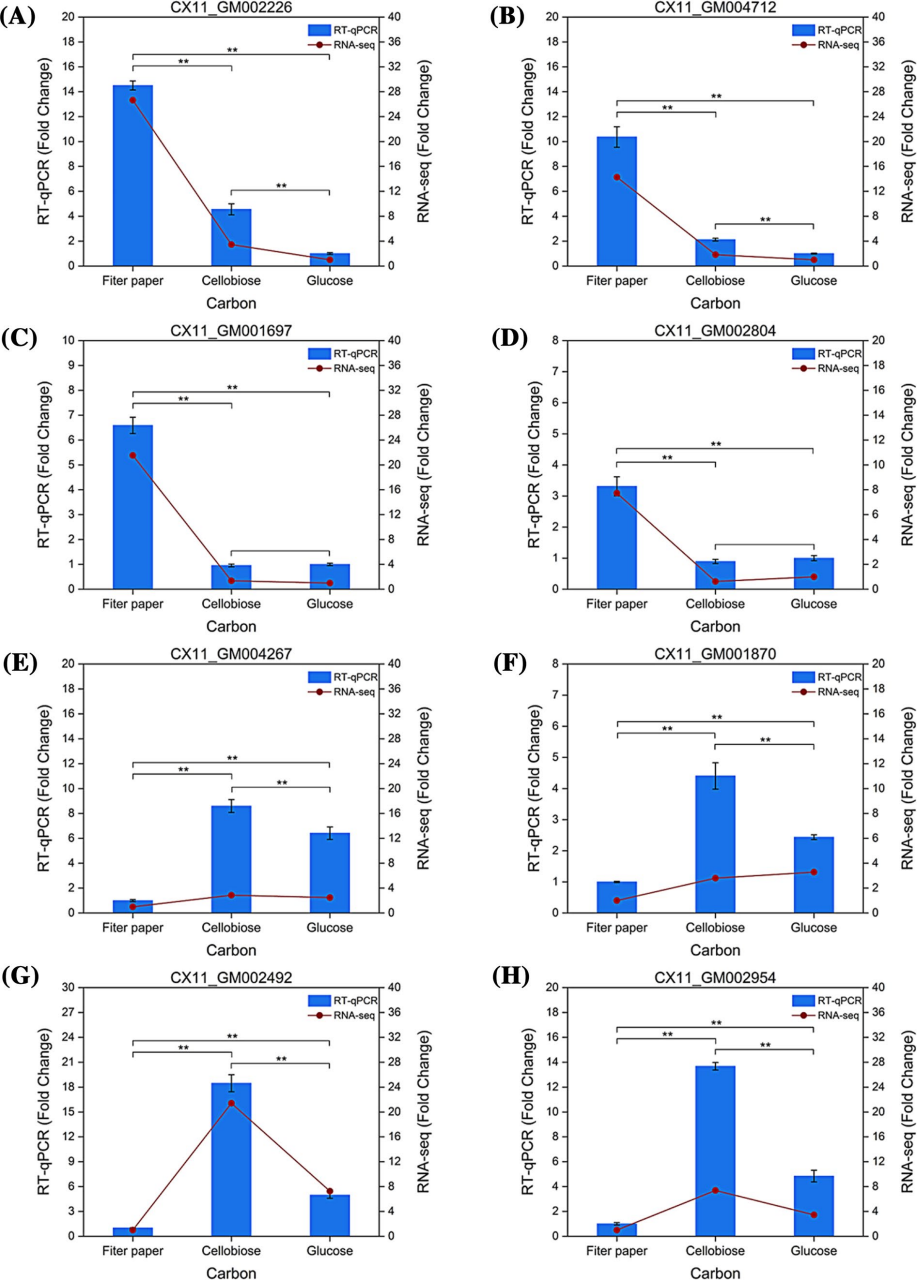
Comparison of RT-qPCR results with RNA-seq results. A and B are the top 2 DEGs in “0” pattern. C and D are the top 2 DEGs in “4” pattern. E and F are the top 2 DEGs in “11” pattern. G and H are the top 2 DEGs in “14” pattern. **means *p* < 0.01, highly significant difference, and no *means no significant difference

RT-qPCR was used to further validate the expression level of genes identified in Illumina sequencing analysis. The top 2 functionally annotated genes in each typical pattern (highlighted in \* MERGEFORMAT Table 2) were chosen as candidates for study (Additional file 1: Table S3). As demonstrated in Fig. 5, these 8 DEGs showed a consistent pattern between Illumina sequencing results and RT-qPCR, indicating that the transcriptome data are reliable and accurate.

### In situ enzymatic activity assays

As shown in Table 1, the 54 significantly upregulated carbohydrate-active enzymes were mainly designated as endoglucanases, xylanases or β-glucosidases. In order to assess the plausibility of the transcriptome analysis and to further identify the biological function of the strain CX11, enzymatic activity assays were performed. The strain was cultured under three different carbon source conditions, and extracellular enzymatic activities were determined.

**Fig. 6:**
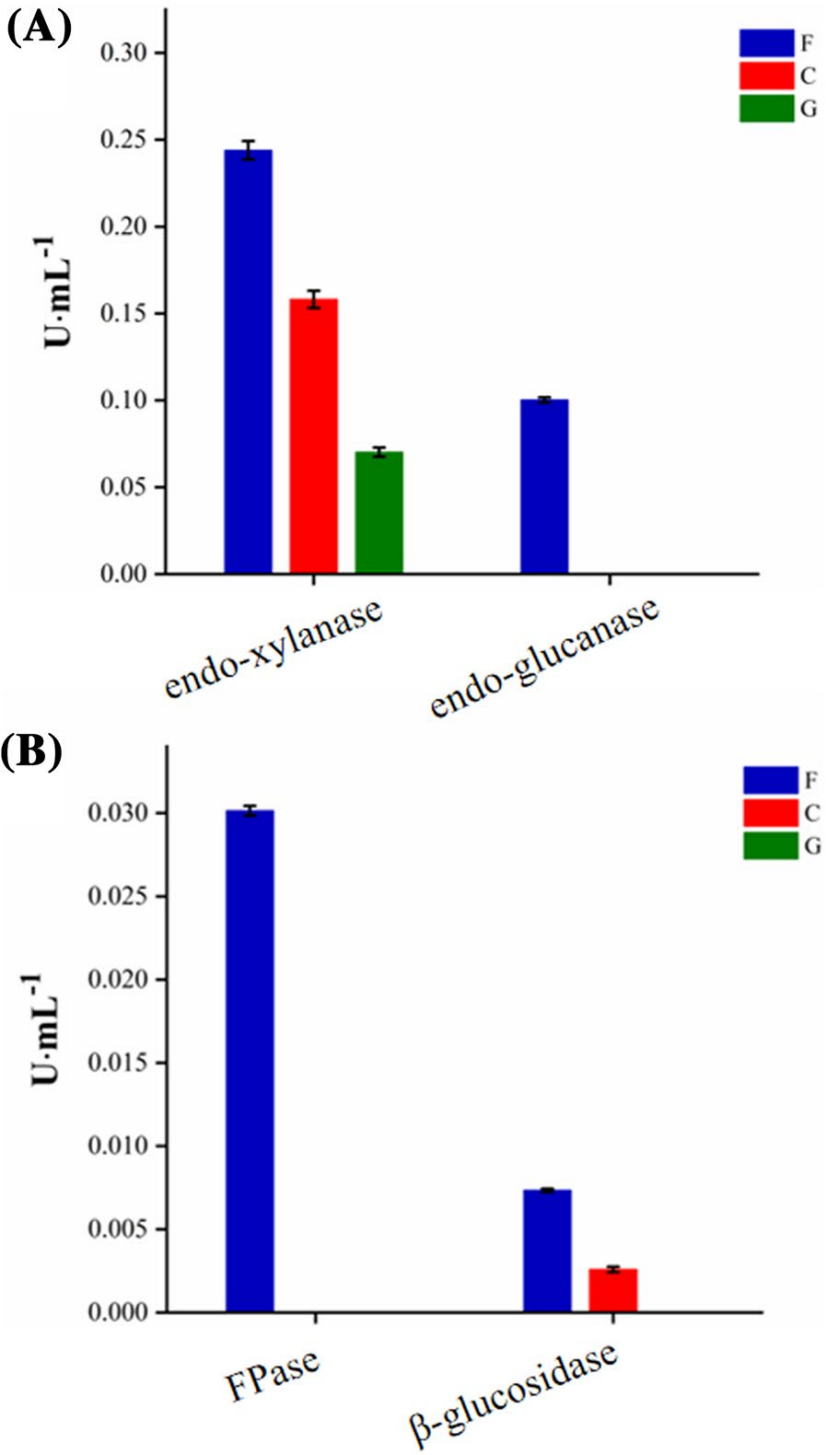
Enzyme activities measured under different carbon source conditions. **A**: Endo-xylanase activity and endo-glucanase activity in fermentation broth under different carbon source conditions. **B**: FPase activity and β-glucosidase activity in fermentation broth under different carbon source conditions. (F/C/G: fermentation supernatant using filter paper, cellobiose and glucose as the sole carbon source, respectively.)

When using filter paper as the sole carbon source, endo-xylanase, FPase, endo-glucanase and β-glucosidase activities were detected in the supernatant of fermentation broth, among which the endo-xylanase activity was the highest. When the strain was cultured with cellobiose as the sole carbon source, endo-xylanase and β-glucosidase activities were identified. Only endo-xylanase could be detected in the broth with glucose as the sole carbon source (Fig. 6). Endo-xylanase activity was detected under all the three different carbon source conditions, indicating that this kind of enzyme might be constitutively synthesized and secreted by strain CX11. While enzymes mainly acting on cellulose are most probably be induced by cellulosic substrates, which is consistent with the results mentioned above that most genes encoding cellulases were upregulated expressed with the existence of filter paper or cellobiose in the culture medium. All these results further confirmed that strain CX11 has a great potential application in the degradation of cellulosic substrates.

## Conclusions

This is the first transcriptomic study of *Sporocytophaga* sp., and many DEGs associated with lignocellulose degradation were identified. These DEGs were involved in multiple GO functional category such as carbohydrate metabolic processes, carbohydrate binding and o-glycosyl compound hydrolase activity. The functional analysis and expression patterns of DEGs, as well as the prediction of functionally unannotated genes related to lignocellulose degradation, lay the foundation for further exploration of biological functions of strain CX11. The results of this study will also offer considerable potential for the application of candidate genes in the utilization of lignocellulose resources.

### Supplementary Information

**Additional file 1: Figure S1.** Histogram of GO statistical results of differentially expressed genes in F vs G group. **Figure S2.** Histogram of GO statistical results of differentially expressed genes in C vs G group. **Figure S3.** Histogram of GO statistical results of differentially expressed genes in F vs C group. **Figure S4.** 16 expression patterns of differentially expressed genes. **Table S1.** Primers of RT-qPCR. **Table S2.** Summary of differentially expressed genes related to gliding function in groups. **Table S3.** Normalized relative expression of candidate genes under different carbon sources.

## Data Availability

All data produced or analyzed for this study are included in the published article and its additional information files.
