# Supplementary material for: Differential transcriptome analysis of Sporocytophaga sp. CX11 and identification of candidate genes involved in lignocellulose degradation
Source: Bioresour Bioprocess. 2023 Jan 30;10(1):8. doi: 10.1186/s40643-023-00629-4 (PMC10992098; doi:10.1186/s40643-023-00629-4)
Supplement: Supplementary file 1 — Additional file 1: Figure S1. Histogram of GO statistical results of differentially expressed genes in F vs G group. Figure S2. Histogram of GO statistical results of differentially expressed genes in C vs G group. Figure S3. Histogram of GO statistical results of differentially expressed genes in F vs C group. Figure S4. 16 expression patterns of differentially expressed genes. Table S1. Primers of RT-qPCR. Table S2. Summary of differentially expressed genes related to gliding function in groups. Table S3. Normalized relative expression of candidate genes under different carbon sources. [file 40643_2023_629_MOESM1_ESM.docx]

**Differential Transcriptome Analysis of *Sporocytophaga* sp. CX11 and Identification of Candidate Genes Involved in Lignocellulose Degradation**

Jiwei Wang^1#^, Ying Zhuang^1#^, Xianghe Song^1^, Xu Lin^1^, Xiangyi Wang^1^, Fan Yang^1^* and Xiaoyi Chen^1^*

^1^ School of Biological Engineering, Dalian Polytechnic University, Ganjingziqu, Dalian 116034, People’s Republic of China

* Correspondence: chen-xy@dlpu.edu.cn (XC) or yang_fan@dlpu.edu.cn (FY)

^1^ School of Biological Engineering, Dalian Polytechnic University, Ganjingziqu, Dalian 116034, People’s Republic of China

^#^ Equal contributors


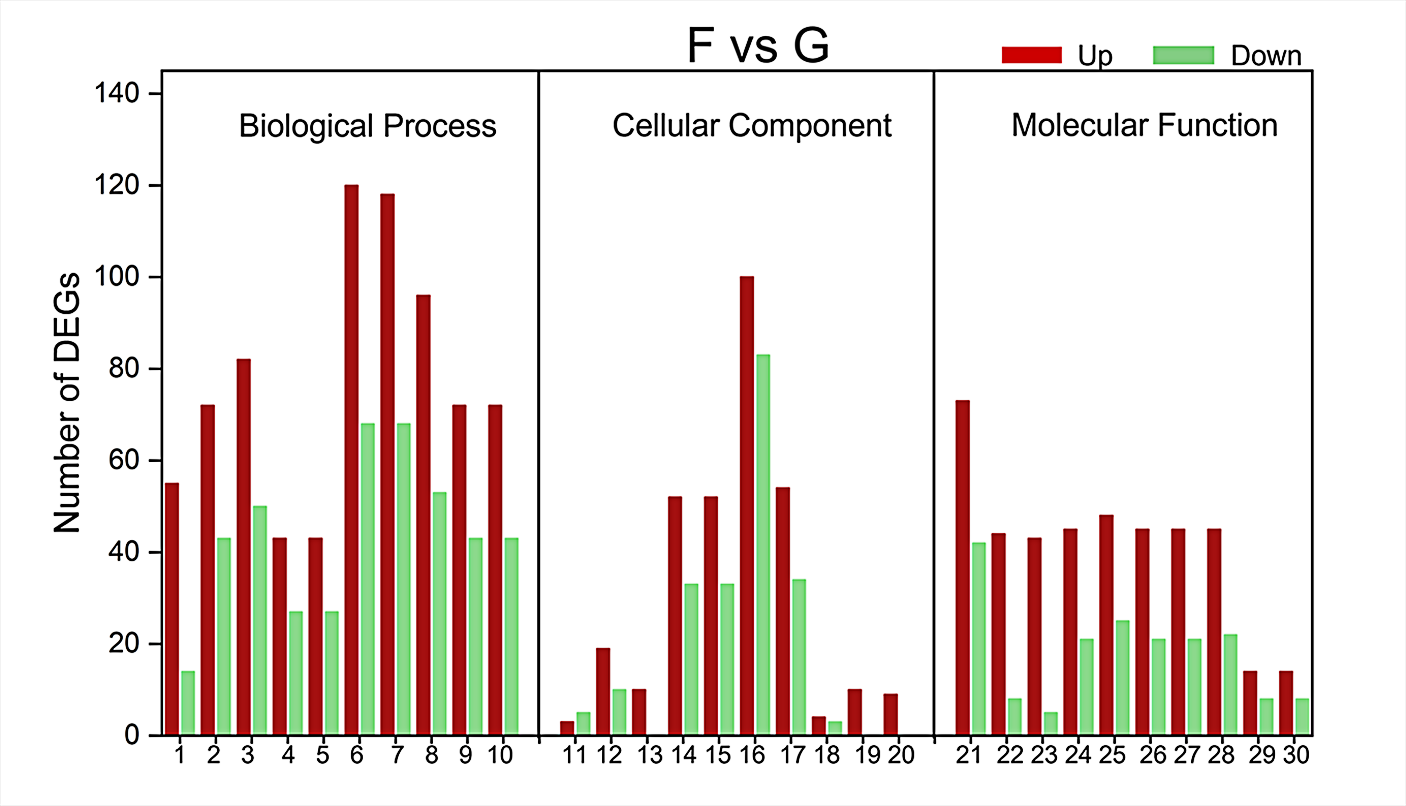


**Fig S1** Histogram of GO statistical results of differentially expressed genes in F vs G group. 1:Carbohydrate metabolic process, 2:Cell communication, 3:Cellular response to stimulus, 4:Intracellular signal transduction, 5:Phosphorelay signal transduction system, 6:Regulation of biological process, 7:Regulation of cellular process, 8:Response to stimulus, 9:Signal transduction, 10:Signal transduction, 11:Cell periphery, 12:Cytoplasm, 13:Cytoplasmic part, 14:Integral component of membrane, 15:Intrinsic component of membrane, 16:Membrane, 17:Membrane part, 18:Membrane protein complex, 19:Ribonucleoprotein complex, 20:Ribosome, 21:Catalytic activity, acting on a protein, 22:Hydrolase activity, acting on glycosyl bonds, 23:Hydrolase activity, hydrolyzing O-glycosyl compounds, 24:Phosphorelay sensor kinase activity, 25:Phosphotransferase activity, alcohol group as acceptor, 26:Phosphotransferase activity, nitrogenous group as acceptor, 27:Protein histidine kinase activity, 28:Protein kinase activity, 29:Serine hydrolase activity, 30:Serine-type peptidase activity.


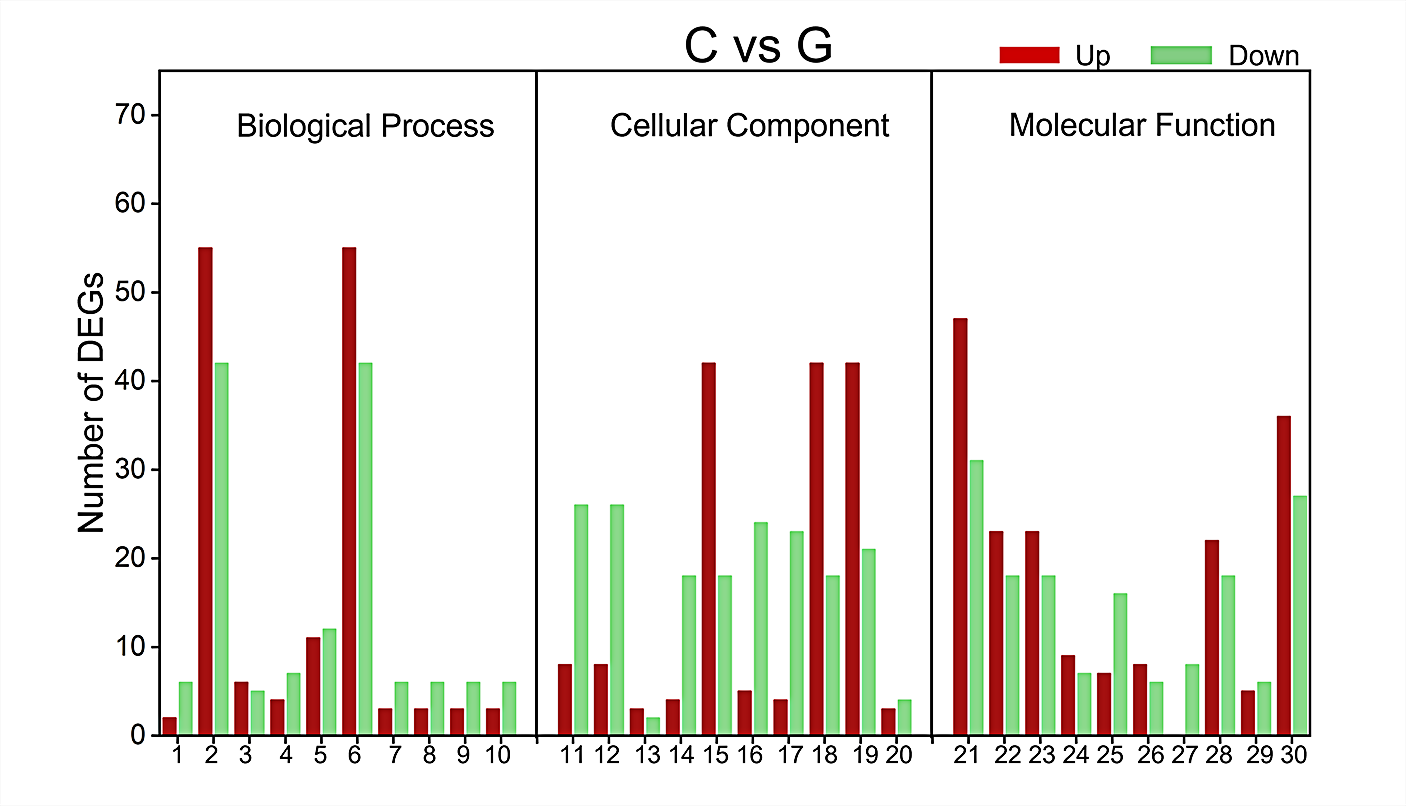


**Fig S2** Histogram of GO statistical results of differentially expressed genes in C vs G group. 1:Aspartate family amino acid metabolic process, 2:Localization, 3:Metal ion transport, 4:Monovalent inorganic cation transport, 5:Proteolysis, 6:Transport, 7:Vitamin biosynthetic process, 8:Vitamin metabolic process, 9:Water-soluble vitamin biosynthetic process, 10:Water-soluble vitamin metabolic process, 11:Cell, 12:Cell part, 13:Cell periphery, 14:Cytoplasm, 15:Integral component of membrane, 16:Intracellular, 17:Intracellular part, 18:Intrinsic component of membrane, 19:Membrane part, 20:Membrane protein complex, 21:Catalytic activity, acting on a protein, 22:Hydrolase activity, acting on acid anhydrides, 23:Hydrolase activity, acting on acid anhydrides, in phosphorus-containing anhydrides, 24:Inorganic cation transmembrane transporter activity, 25:Isomerase activity, 26:Monovalent inorganic cation transmembrane transporter activity, 27:Oxidoreductase activity, acting on the CH-CH group of donors, 28:Pyrophosphatase activity, 29:Serine-type endopeptidase activity, 30:Transporter activity.


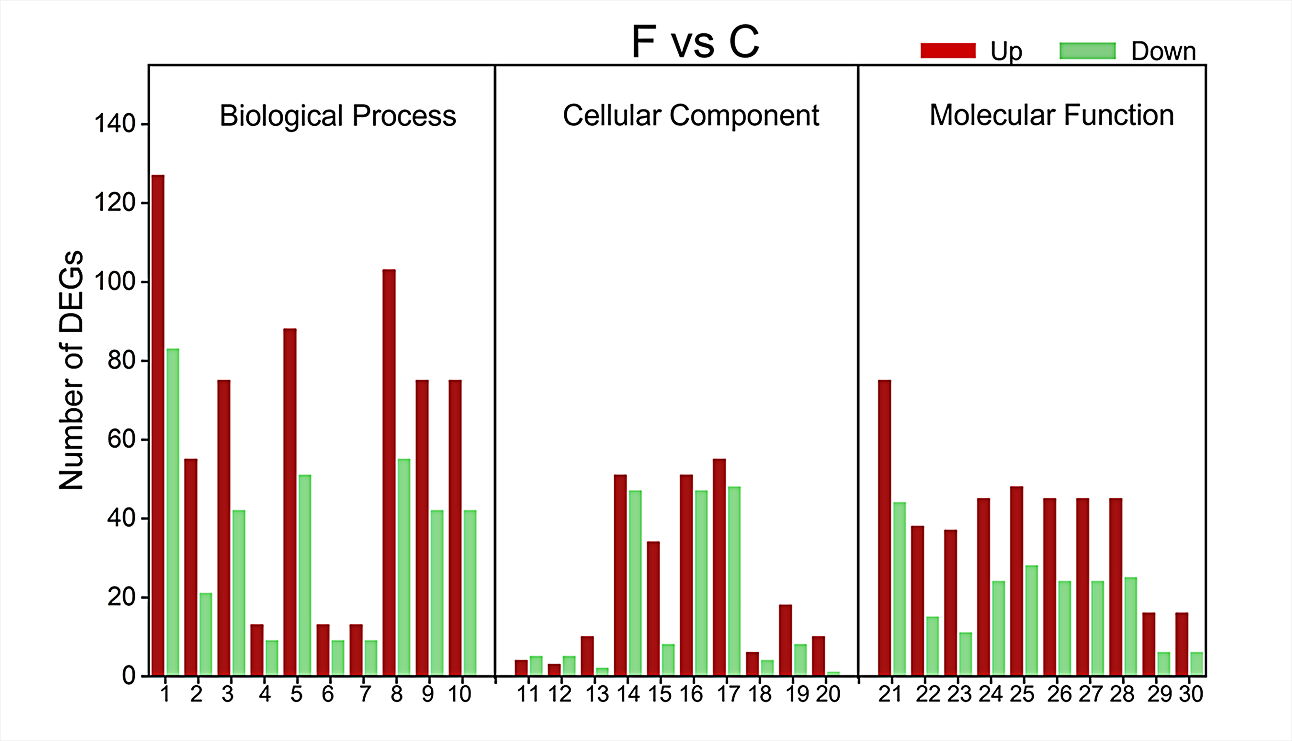


**Fig S3** Histogram of GO statistical results of differentially expressed genes in F vs C group. 1:Biological regulation, 2:Carbohydrate metabolic process, 3:Cell communication, 4:Cellular response to DNA damage stimulus, 5:Cellular response to stimulus, 6:Cellular response to stress, 7:DNA repair, 8:Response to stimulus, 9:Signal transduction, 10:Signal transduction, 11:Catalytic complex, 12:Cell periphery, 13:Cytoplasmic part, 14:Integral component of membrane, 15:Intracellular part, 16:Intrinsic component of membrane, 17:Membrane part, 18:Membrane protein complex, 19:Protein-containing complex, 20:Ribonucleoprotein complex, 21:Catalytic activity, acting on a protein, 22:Hydrolase activity, acting on glycosyl bonds, 23:Hydrolase activity, hydrolyzing O-glycosyl compounds, 24:Phosphorelay sensor kinase activity, 25:Phosphotransferase activity, alcohol group as acceptor, 26:Phosphotransferase activity, nitrogenous group as acceptor, 27:Protein histidine kinase activity, 28:Protein kinase activity, 29:Serine hydrolase activity, 30:Serine-type peptidase activity.


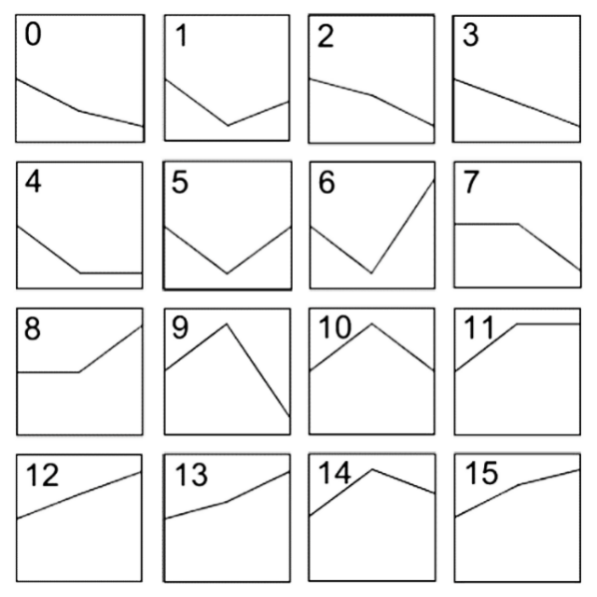


Fig S4 16 expression patterns of differentially expressed genes

Table S1 Primers of RT-qPCR

| 基因名称 | 引物序列（5'-3'） |
| --- | --- |
| *CX11_GM002226* | F：CGGATTGCAGGCTACTTATG |
|  | R：GGTTCAGATGTCCCACCAATA |
| *CX11_GM004712* | F：GCCTCTCCGAATGGCAATAA |
|  | R：CTCAACCTCAGTGCTGGTAAA |
| *CX11_GM001697* | F：AGGAAGCAGGGAAGCAATC |
|  | R：CGGCATATCTCCACTCAATGT |
| *CX11_GM002804* | F：TTCGGAGCAGAAGAGACTAATG |
|  | R：CGGAAACCGGAAGTAAGATAGA |
| *CX11_GM004267* | F：GTTGATGCGGCAAGGAAATTAG |
|  | R：GTGGTAAGTGCTGATGGTGAA |
| *CX11_GM001870* | F：TGTCTGCTGGTGCGATATAC |
|  | R：CATTAGGAGACGATAAGTGGTAGG |
| *CX11_GM002954* | F：TCCGATAAGGAGTGGCAGTG |
|  | R：CGGTAATCCCAAGAGTAGTT |
| *CX11_GM002492* | F：CACTTCTCTCGCTCTTGATAG |
|  | R：GTAGTTCCAAAAGCCAGTTG |

Table S2 Summary of differentially expressed genes related to gliding function in groups

|  |  | log_2_ Fold Change | | |
| --- | --- | --- | --- | --- |
| Gene ID | Gene description | F vs G | C vs G | F vs C |
| *CX11_GM000212* | Gliding motility protein GldM | 2.28 | -0.12 | 2.28 |
| *CX11_GM000815* | Gliding motility protein GldM | 2.91 | -0.49 | 3.29 |
| *CX11_GM004043* | Gliding motility lipoprotein GldH | -1.14 | 0.56 | -1.80 |
| *CX11_GM000076* | Type IX secretion system membrane protein PorP/SprF | -1.63 | -0.28 | -1.44 |
| *CX11_GM000085* | Type IX secretion system membrane protein PorP/SprF | -1.43 | -0.42 | -1.10 |
| *CX11_GM000183* | Type IX secretion system membrane protein PorP/SprF | -1.01 | -0.13 | -1.00 |
| *CX11_GM000238* | Type IX secretion system membrane protein PorP/SprF | -1.92 | 1.14 | -3.20 |
| *CX11_GM000812* | Type IX secretion system membrane protein PorP/SprF | 0.47 | -0.94 | 1.30 |
| *CX11_GM001408* | Type IX secretion system membrane protein PorP/SprF | -3.01 | 0.00 | -3.11 |
| *CX11_GM001697* | Type IX secretion system membrane protein PorP/SprF | 4.35 | 0.31 | 3.95 |
| *CX11_GM001870* | Type IX secretion system membrane protein PorP/SprF | -1.69 | -0.28 | -1.50 |
| *CX11_GM001892* | Type IX secretion system membrane protein PorP/SprF | -1.23 | 0.00 | -1.32 |
| *CX11_GM002029* | Type IX secretion system membrane protein PorP/SprF | 1.92 | 0.65 | 1.16 |
| *CX11_GM002226* | Type IX secretion system membrane protein PorP/SprF | 4.74 | 1.70 | 2.93 |
| *CX11_GM002369* | Type IX secretion system membrane protein PorP/SprF | -0.75 | -0.13 | -0.72 |
| *CX11_GM002698* | Type IX secretion system membrane protein PorP/SprF | 2.13 | -0.04 | 2.08 |
| *CX11_GM003048* | Type IX secretion system membrane protein PorP/SprF | -0.53 | 0.34 | -0.97 |
| *CX11_GM003265* | Type IX secretion system membrane protein PorP/SprF | -1.11 | 0.20 | -1.40 |
| *CX11_GM003958* | Type IX secretion system membrane protein PorP/SprF | -1.89 | -0.44 | -1.52 |
| *CX11_GM004089* | Type IX secretion system membrane protein PorP/SprF | 0.71 | 0.48 | 0.13 |
| *CX11_GM004293* | Type IX secretion system membrane protein PorP/SprF | -1.55 | -0.58 | -1.08 |
| *CX11_GM004315* | Type IX secretion system membrane protein PorP/SprF | -1.09 | -0.38 | -0.81 |
| *CX11_GM005032* | Type IX secretion system membrane protein PorP/SprF | -2.02 | 0.29 | -2.41 |
| *CX11_GM005390* | Type IX secretion system membrane protein PorP/SprF | -0.80 | 0.66 | -1.53 |
| *CX11_GM005434* | Type IX secretion system membrane protein PorP/SprF | 1.22 | 0.98 | 0.14 |
| *CX11_GM005668* | Type IX secretion system membrane protein PorP/SprF | -0.64 | -0.75 | 0.01 |
| *Novel00976* | Type IX secretion system membrane protein PorP/SprF | 4.01 | 2.33 | 1.60 |
| *CX11_GM000741* | SprT-like family | 1.48 | -0.46 | 1.82 |
| *CX11_GM001013* | SprB repeat | -1.29 | 0.54 | -1.93 |
| *CX11_GM001117* | SprB repeat | 1.73 | 0.82 | 0.80 |
| *CX11_GM001485* | SprB repeat | -0.04 | 0.68 | -0.83 |
| *CX11_GM001881* | SprB repeat | -0.60 | -0.09 | -0.61 |
| *CX11_GM002027* | SprB repeat | 2.94 | 0.58 | 2.25 |
| *CX11_GM003640* | SprB repeat | -0.47 | 0.86 | -1.44 |
| *Novel00303* | SprB repeat | -2.72 | 0.44 | -3.25 |
| *CX11_GM001407* | TonB-dependent receptor SusC | -1.81 | 0.35 | -2.28 |
| *CX11_GM004445* | TonB-dependent receptor SusC | 3.10 | 0.65 | 2.34 |
| *CX11_GM004127* | SusD family | -1.07 | 0.36 | -1.54 |
| *CX11_GM004444* | SusD family | 2.19 | 0.37 | 1.72 |

Table S3 Normalized relative expression of candidate genes under different carbon sources

| Gene ID | Filter paper | Cellobiose | Glucose |
| --- | --- | --- | --- |
| *CX11_GM002226* | 14.5 | 4.56 | 1 |
| *CX11_GM004712* | 10.37 | 2.12 | 1 |
| *CX11_GM001697* | 6.59 | 0.96 | 1 |
| *CX11_GM002804* | 3.31 | 0.89 | 1 |
| *CX11_GM004267* | 1 | 8.6 | 6.42 |
| *CX11_GM001870* | 1 | 4.41 | 2.44 |
| *CX11_GM002492* | 1 | 18.47 | 4.96 |
| *CX11_GM002954* | 1 | 13.68 | 4.84 |

**Method of detecting enzyme activity**

For the detection of endo-1,4-beta-glucanase and xylanase activities, the reaction mixture contained 50 μL of fermentation broth and 1% (w/v) of CMC-Na or xylan in 10 mM HAc−NaAc buffer (pH 5.5). The reaction was performed at 30 ℃ for 20 min. For measuring the FPase, the reaction mixture contained 50 μL of fermentation broth and 5% (w/v) of Whatman No.1 filter paper in 10 mM HAc−NaAc buffer (pH 5.5). The reaction was performed at 30 ℃ for 60 min. The released reducing sugars were measured using 3, 5-dinitrosalicylic acid (DNS) as previously described (Miller 1959).

To measure β-glucosidase activity, 250 μL of fermentation broth was mixed with 250 μL of 1.25 mM *p*NPG in 10 mM HAc−NaAc buffer (pH 5.5). The reaction was performed at 30 ℃ for 30 min and stopped by adding 1 mL of 1 M Na_2_CO_3_. The released *p*NP was monitored at 405 nm by SpectraMax Paradigm Multi-Mode detection platform (Molecular Devices, Sunnyvale, CA).
